# Improving the Performance of a Ballistic Protection Composite with Either Graphene Oxide or Molybdenum Disulfide

**DOI:** 10.1021/acsomega.5c07636

**Published:** 2025-11-12

**Authors:** Josué Marciano de Oliveira Cremonezzi, Gabriel Matheus Pinto, Natália Nascimento Pereira, Rosica Mincheva, Ricardo Jorge Espanhol Andrade, Jean-Marie Raquez, Guilhermino José Macedo Fechine

**Affiliations:** † MackGraphe−Mackenzie Institute for Graphene and Nanotechnology, Rua da Consolação, 896, Consolação, São Paulo, SP 01302-907, Brazil; ‡ School of Engineering, 42524 Mackenzie Presbyterian University, Rua da Consolação, 896, Consolação, São Paulo, SP 01302-907, Brazil; § Inbrafiltro, Av. Papa João XXIII, 4947, Vila Noemia, Mauá, SP 09370-800, Brazil; ∥ Laboratory of Polymeric and Composite Materials, Center of Innovation and Research in Materials and Polymers (CIRMAP), University of Mons, Place du Parc 20, 7000 Mons, Belgium

## Abstract

This study investigates the ballistic performances of aramid composites reinforced with either graphene oxide (GO) or molybdenum disulfide (MoS_2_), two-dimensional (2D) nanomaterials known for their exceptional mechanical properties and large specific surface areas. Hierarchical composites were developed by incorporating these nanomaterials into the polymeric matrix and/or depositing them onto the Kevlar fiber surface, and their performance was compared to conventional Kevlar/PVB composites. Dynamic mechanical analysis revealed increased storage modulus and improved fiber/matrix interfacial adhesion, contributing to tensile toughness gains of up to 90.3% over the unmodified composite. Fractographic analysis confirmed strong interactions between Kevlar fibers and the nanomodified matrices. Ballistics tests conducted on level II-A body armor prototypes showed reduced back face signature and enhanced impact resistance, with higher specific absorbed energy and ballistic limit than the reference composite. These findings highlight the potential of nanomodified hierarchical composites for next-generation body armor applications.

## Graphical abstract

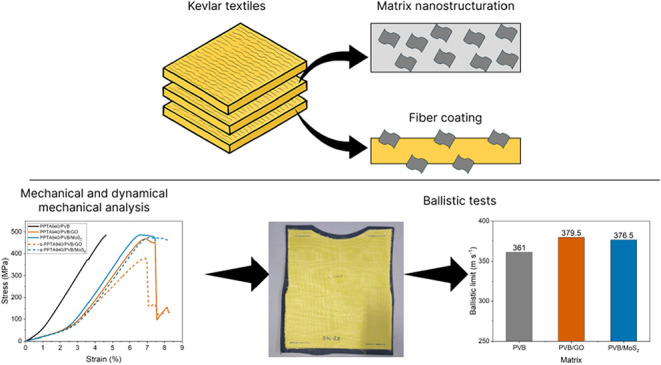

## Introduction

Throughout history, humanity has faced external threats from the environment, enemies, and animals. Territorial expansion among distinct groups in search of food, resources, and wealth has inevitably led to disputes and conflicts, often involving the use of force. These conflicts continue to this day. As a result, weaponry has been continuously developed to increase firepower. Consequently, armor and other defensive systems have also evolved to provide protection against new threats.^1,2^

Armor systems are designed to shield bodies or objects from being penetrated by weapons, projectiles, shrapnel, or blast fragments. This protective gear is critical for the survival and effectiveness of military and police forces in the field, as well as civilians like security personnel, influential figures, and journalists in conflict zones. However, although research shows that the heavy weight of such equipment can significantly diminish individual effectiveness,^3^ the current protective equipment remains far from lightweight. The American military bulletproof vest, considered as the world’s most advanced, weighs approximately 4.8 kg and can increase to 12.1 kg when adapted for higher-caliber protection. When combined with additional gear, the total equipment weight can reach up to 30 kg.^3^ To enhance wearability and user mobility, the design of body armor must prioritize the flexibility and lightness of materials, while maximizing energy absorption efficiency to mitigate the physiological stress caused by projectile impacts.^1^

Multiple energy absorption mechanisms simultaneously decelerate and ideally retain the projectile.^2,4–6^ The main mechanisms are based on fiber stretching and fracture, and composite delamination, with the energy being directly proportional to the fiber/matrix adhesion.^5,7,8^ Thus, enhancing the performance of fiber-reinforced polymer composites (FRPCs) requires not only optimizing the fiber architecture^9^ and matrix properties but also improving stress transfer and energy dissipation mechanisms. Building on these mechanisms, the incorporation of nanofillers has emerged as a promising strategy to further enhance the mechanical performance and energy absorption capabilities of FRPCs.^10^ Some authors^4,11–16^ have shown that nanofillers in the matrix or at the fiber/matrix interface can enhance stress transfer, delay crack propagation, and promote additional energy dissipation mechanisms.

Nanomaterials of different structures and chemical composition^17–20^ such as graphene derivates, carbon nanotubes and inorganic nanotubes, fullerene like nanomaterials, among others have been vastly considered. However, despite these promising results, the extent of improvement varies considerably depending on the type of filler, its dispersion, and the processing route and a lack of comparison between nanomaterials and incorporation strategies still remain.

Moreover, although aramid impregnated with poly­(vinyl butyral) (PVB) is widely used in commercial ballistic systems due to its favorable balance between flexibility, toughness, and adhesion, this specific configuration has received comparatively little attention in scientific literature. As a result, there remains a knowledge gap regarding how nanofillers can be effectively integrated into Kevlar/PVB systems to optimize their mechanical and ballistic performance.

Two-dimensional (2D) materials, such as graphene derivatives and transition-metal dichalcogenides (TMD), are attractive candidate polymer reinforcing fillers because of their high specific surface area, remarkable mechanical properties and tunable surface chemistry. These features can enhance interfacial adhesion, promote load transfer, and introduce distinct dissipation mechanisms such as nanosheet pull-out/peel and crack deflection, even at low loadings.^21^ Graphene, a single layer of carbon atoms arranged in a hexagonal lattice, is renowned for its extraordinary tensile strength of up to 130 GPa and a Young’s modulus of approximately 1 TPa.^22^ Graphene oxide (GO), a modified form of graphene, maintains much of graphene’s mechanical strength, while offering improved chemical interactions with polymer due to its oxygen-containing functional groups.^23,24^ Molybdenum disulfide (MoS_2_), a layered transition-metal dichalcogenide, exhibits high mechanical strength, with a Young’s modulus of approximately 270 GPa and tensile strength around 23 GPa.^25,26^ In addition to its stiffness, its intrinsic lubricity favors distinct interfacial reinforcement and energy dissipation mechanisms, making it a promising nanofiller for polymer composites.^27–29^ In a previous investigation,^30^ GO and MoS_2_ were shown to exhibit distinct reinforcement behaviors when incorporated into neat PVB, highlighting their divergent interaction pathways with the polymer. This raises an open question of whether such differences remain relevant when these nanocomposites are used as matrices in Kevlar-based hierarchical composites subjected to both quasi-static deformation and ballistic impact.

Therefore, a systematic study that compares GO and MoS_2_ in both matrix and surface-coating configurations, and that combines micromechanical characterization with standardized ballistic trials, is required to establish whether their chemical, structural, and morphological differences translate into distinct reinforcement effects. Here, we address this gap exploring a strategy to enhance aramid FRPC performance by incorporating either GO or MoS_2_ into a PVB matrix and/or as a coating to the fiber surface. The reinforcement effects were assessed by evaluating their micromechanical, dynamic-mechanical, and ballistic performance. This approach enabled a direct comparison of (i) the chemical interfacial effects of GO, (ii) the morphological/dissipative contribution of MoS_2_ nanosheets, and (iii) the relative effectiveness of matrix nanostructuration versus fiber coating strategies in a realistic armor configuration.

## Experimental Section

### Materials

Kevlar fabrics employed in this work had linear yarn density of 940 dtex. Solutions of PVB, PVB/GO, and PVB/MoS_2_ nanocomposites with 0.50 wt % filler were prepared as described elsewhere.^30^

### Sample Preparation

Figure 1a shows an illustrated schema of the main steps of the samples’ preparation. Kevlar textiles were cut into 250 mm × 250 mm squares with electric scissors and oven-dried at 100 °C for 1 h to remove moisture. The fabrics were weighed and divided into two groups: untreated (“PPTA”) and surface-modified (“c-PPTA”). PPTA refers to poly­(*p*-phenylene terephthalamide), the chemical structure of Kevlar. For surface modification, “c-PPTA” fabrics underwent dip-coating in ethanol dispersions of GO or MoS_2_ (1.0 mg mL^–1^) for 24 h at room temperature, followed by drying in a vacuum oven at 100 °C for 1 h.

**Figure 1:**
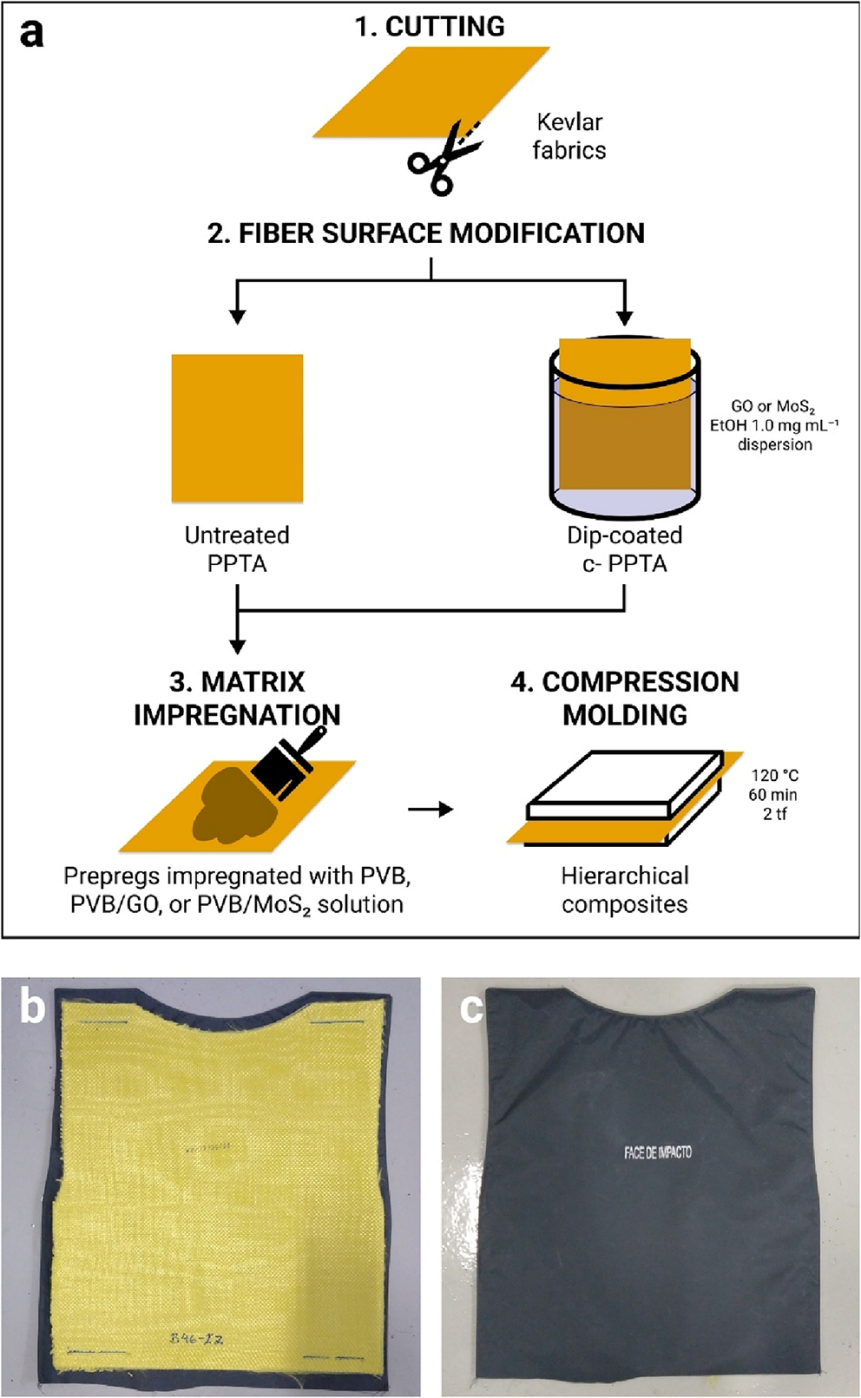
(a) Illustrative schema of the main steps of the hierarchical composites’ preparation method; and digital photographs of (b) a dorsal armor plaque specimen and (c) the nylon cover.

Prepregs with the compositions summarized in Table 1 were prepared by hand layup. Approximately 25–30 g of the respective matrix solution (PVB, PVB/GO, or PVB/MoS_2_) was spread on one side of the fabric with a plastic-toothed spatula, dried for 15 min at room temperature, and then applied to the opposite side in the same way. The impregnated fabrics were dried in a vacuum oven at 80 °C for 15 min.

**Table 1:** Composition and Nomenclature of the Hierarchical Composites Produced

fabric	matrix	nomenclature
PPTA	PVB	PPTA/PVB
PPTA	PVB/GO 0.50 wt %	PPTA/PVB/GO
PPTA	PVB/MoS_2_ 0.50 wt %	PPTA/PVB/MoS_2_
PPTA covered by GO	PVB/GO 0.50 wt %	c-PPTA/PVB/GO
PPTA covered by MoS_2_	PVB/MoS_2_ 0.50 wt %	c-PPTA/PVB/MoS_2_

The prepregs were consolidated into hierarchical composites by compression molding. They were placed between steel plates covered with Teflon films and pressed at 120 °C for 60 min under a load of 2 tf, followed by cooling to room temperature for 7 min. The resulting composites were weighed.

Tapes were cut from the fabrics for tensile testing and dynamic mechanical thermal analysis (DMA). The tensile specimens measured 240 mm × 15 mm, and the DMA specimens 30 mm × 8 mm, both cut with electric scissors.

For the manufacture of armor plaques, Kevlar textiles were also cut into 500 mm × 500 mm squares using an automated cutting machine and impregnated with PVB, PVB/GO, or PVB/MoS_2_, as described above and dried at ambient temperature. For each plaque, 8 Kevlar prepregs were sandwiched between two X-Flex Ultra 304 Kevlar textiles, cut into the shape of the GG EP-149 dorsal vest, and sewn together (Figure 1a). The plaques were placed inside nylon covers for ballistic testing (Figure 1b). Two specimens of each composite were produced.

### Characterization Methods

SEM was performed using a JSM-6510 (Jeol) scanning electron microscope with a secondary electron detector and an acceleration voltage of 30 kV. Before imaging, the samples were coated with gold via sputtering. Image processing was performed using the public-domain software ImageJ 1.53k (National Institutes of Health).

Fourier transform infrared spectroscopy (FTIR) was performed in an IRAffinity-1S (Shimadzu) infrared spectrometer. The reported spectra are the mean of three spectra acquired in 128 scans with a resolution of 2 cm^–1^.

Dynamic mechanical analysis was conducted in a DMA Q800 (TA Instruments) thermal analyzer in tensile mode. The temperature range was −10 to 175 °C, with a ramp of 5 °C min^–1^. The frequency was 1 Hz, and the strain was 0.1%.

The tensile test was conducted using a ProLine universal tensile testing machine (ZwickRoell). ASTM D 6775–02 and ASTM D 3039 served as guidelines. The distance between the grips was 160 mm, and the crosshead speed was 75 mm min^–1^. A load cell with a capacity of 2.5 kN was utilized.

Ballistics tests were conducted at InbraFiltro Ind. e Com. de Filtros LTDA facility following the NIJ 0101.04 standard^31^ for II-A body armor. At least six projectiles were fired at each specimen using a proprietary air-powered test weapon. The ammunition consisted of 9 mm full metal jacket projectiles (Companhia Brasileira de Cartuchos) with the gunpowder charge adjusted to reach the desired velocities, a standard method in the ballistic plate manufacturing industry to achieve precision and reproducibility. The impact velocity of each projectile was recorded using a chronograph. The first three shots in each specimen were within the velocity range specified by the standard, while subsequent shots had increasing velocities to determine the ballistic limit of each composite. After the tests, the number of perforated Kevlar plies was assessed by visual inspection, and the back face signature was measured on the plasticine witness panel.

### Data Analysis

Measurements are reported with a 95% confidence interval whenever feasible. Relevant data were subjected to one-way analysis of variance (ANOVA) and Tukey’s Honestly Significant Difference (HSD) post hoc test, maintaining a simultaneous confidence level of 95%. All statistical analyses were performed using Minitab 17 (Minitab).

## Results and Discussion

### Covering of Fibers

The aspect of the fibers covered with GO and MoS_2_ can be observed in the SEM images shown in Figure 2. Although the neat fiber presented some agglomerated particles and minor imperfections on their surface (Figure 2a,b), the fabrics that were immersed in the dispersions noticeably had GO nanosheets (Figure 2c,d) and small MoS_2_ flakes (Figure 2e,f) covering the fibers’ surface, as indicated by yellow arrows in Figure 2.

**Figure 2:**
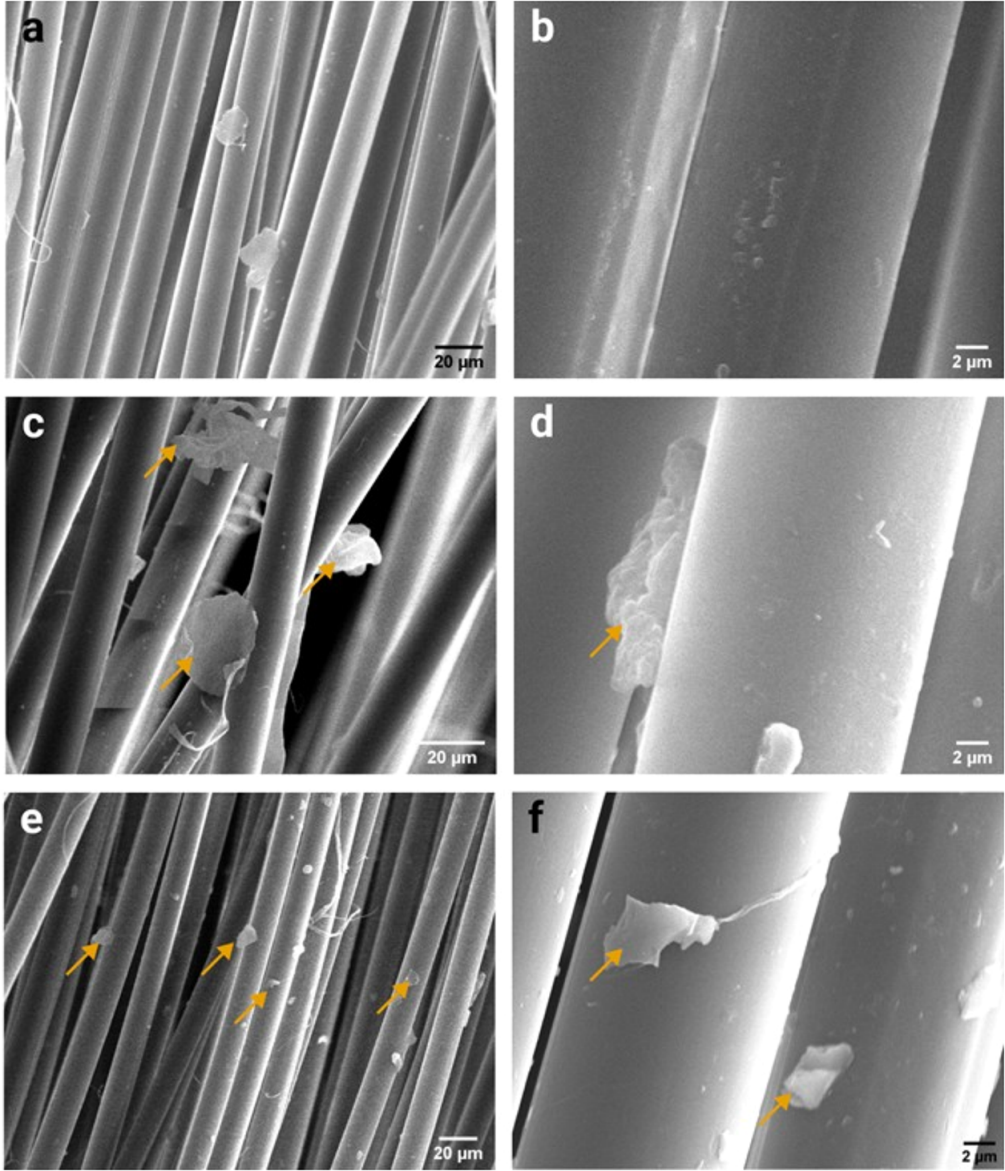
SEM images of (a, b) PPTA, (c, d) GO-covered PPTA, and (e, f) MoS_2_-covered PPTA. Yellow arrows indicate possible nanofillers particles deposited on the fibers surface.

Previous studies have demonstrated that aramid fibers can interact effectively with GO nanosheets through noncovalent mechanisms such as π–π stacking and hydrogen bonding.^32,33^ On the other hand, there is no evidence that MoS_2_ interacts with the aromatic rings of aramid fibers unless one of them has been chemically functionalized.^34,35^ To investigate these interactions in the present work, FTIR analysis was performed on coated fibers. As shown in Figure 3, the FTIR spectra of the coated fibers appeared as a cumulative spectrum of the neat fiber^36^ and the nanomaterials.^37–42^ The band at 3315 cm^–1^ was assigned to the stretching vibration of the aramid N–H bonds. The band at 1635 cm^–1^ was assigned to the amide C=O stretching. The N–H bending vibration and C–N stretching coupled modes gave rise to the band at 1543 cm^–1^. The C=C stretching of the aromatic groups appeared as a shoulder in the same band at 1515 cm^–1^. The bands at 1018 and 827 cm^–1^ were assigned to the in-plane and out-of-plane C–H stretching vibration, respectively. The band at 720 cm^–1^ was assigned to the out-of-plane deformation mode of the N–H bonds.^36^ The weak absorptions at 2925 and 2850 cm^–1^ showed in the GO- and MoS_2_-covered PPTA spectra may have resulted from arise from residual ethanol present in the nanofillers as the result of the exfoliation^38^ and/or in the fibers surface as result of the solution dip-coating. However, the absence of additional or shifted peaks in the nanofiller-coated fibers, no specific interactions could be detected. This result suggests that, although molecular interactions may occur, they were not detectable via FTIR, likely due to their noncovalent nature and the relatively low concentration or sensitivity limitations of the technique.

**Figure 3:**
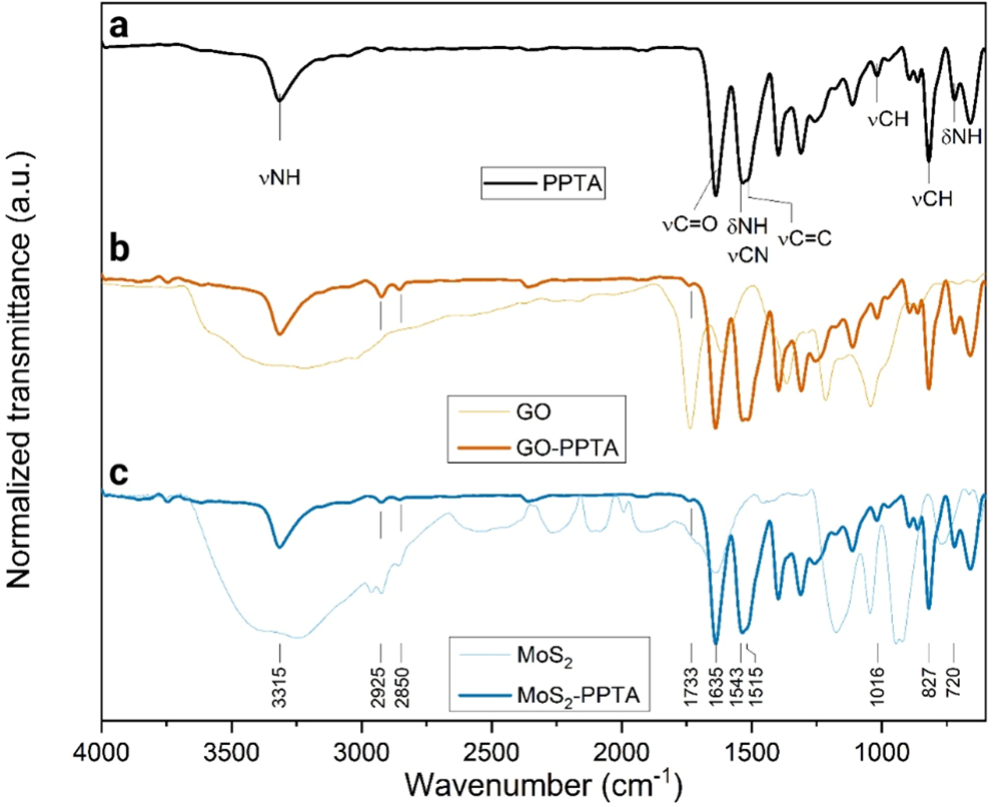
FTIR spectra of (a) PPTA, (b) GO-covered PPTA, and (c) MoS_2_-covered PPTA.

### Dynamic Mechanical Analysis

The reinforcement effects of the nanofillers in the hierarchical composites were initially assessed through DMA. Figure 4a illustrates the storage moduli (*E*′) of the PPTA composites. Both PPTA/PVB/GO and PPTA/PVB/MoS_2_ hierarchical composites showed higher *E*′ values compared to PPTA/PVB at the glassy state (0 °C), with increases of 22 and 29%, respectively. The hierarchical composites consistently maintained higher *E*′ values than PPTA/PVB throughout the entire temperature range, indicating the effective reinforcement from the nanomodified matrices. This enhancement can be attributed to several factors: (i) the high-aspect-ratio 2D nanomaterials provide efficient stress transfer within the polymer matrix; (ii) the presence of nanofillers restricts polymer chain mobility, thereby increasing matrix stiffness; and (iii) improved filler/matrix interactions reduce localized deformation and help distribute mechanical loads more uniformly across the composite.^43,44^ Collectively, these mechanisms contribute to the observed pronounced increase in *E*′, effectively reinforcing the hierarchical composites without requiring modifications to the fiber architecture.

**Figure 4:**
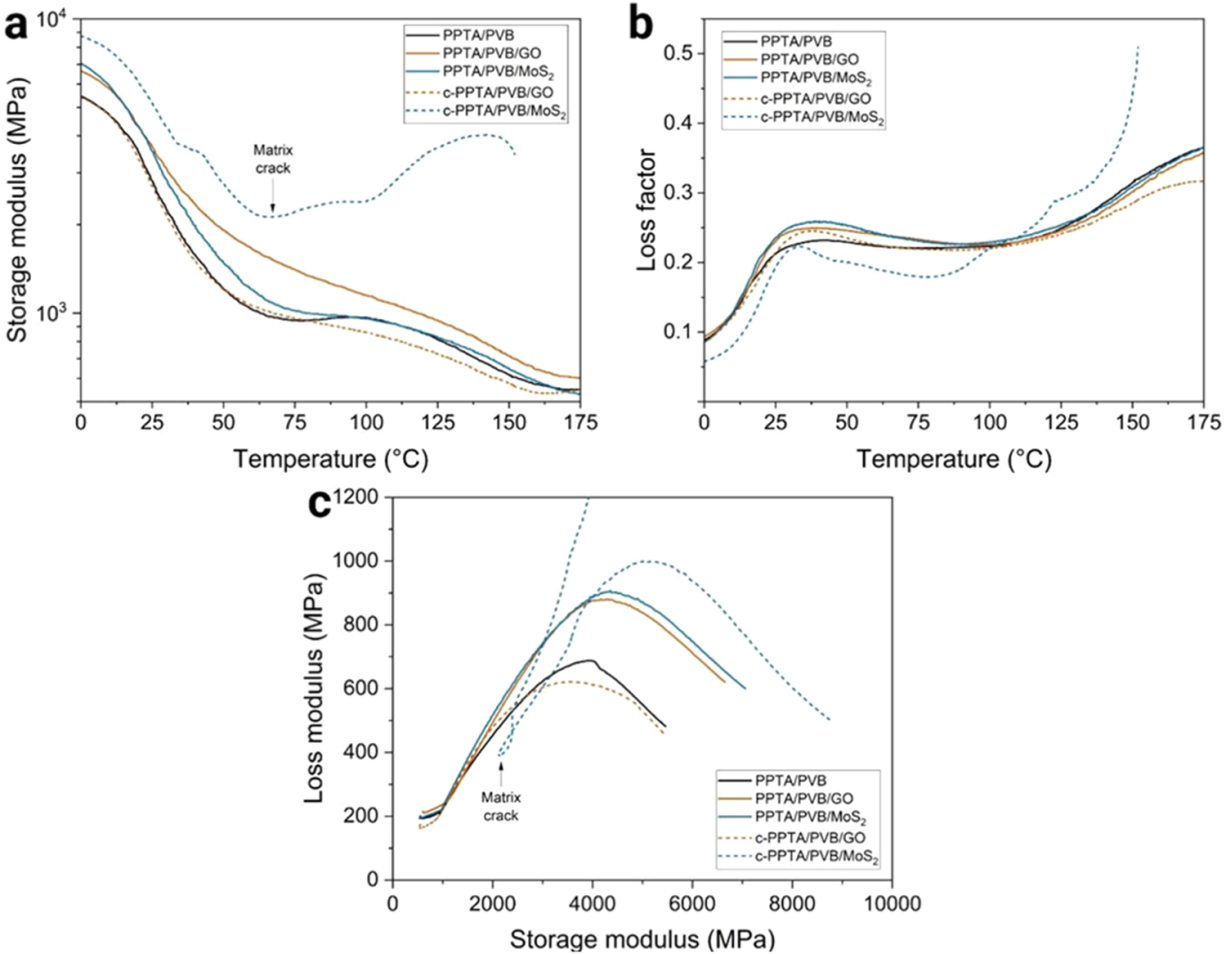
DMA curves of the hierarchical composite tapes: (a) storage modulus, (b) loss factor, and (c) Cole–Cole plot.

From a functional standpoint, such increases in *E*′ are promising in the context of ballistic applications, where enhanced energy absorption is critical. For instance, Obradović et al.^20^ reported that a modest 3% increase in the glassy state *E*′ of an aramid armor with PVB matrix modified with 1.0% of IF-WS_2_ led to 18 and 33% increases in absorbed energy under Charpy impact and knife penetration tests, respectively. Therefore, the significantly higher *E*′ improvement observed for PPTA/PVB/GO and PPTA/PVB/MoS_2_ composites suggest that even greater gains in impact performance could be expected.

In contrast, composites with nanofiller-coated fibers deviated from the trend. The c-PPTA/PVB/GO composite showed no notable improvement in *E*′ compared to PPTA/PVB, indicating that GO coating did not contribute effectively to reinforcement. This may be attributed to weak interfacial interactions or poor dispersion of the filler at the fiber’s surface, as shown in SEM images, limiting load transfer. Conversely, the c-PPTA/PVB/MoS_2_ composite displayed a markedly higher *E*′ at low temperature but exhibited an atypical drop in modulus at elevated temperatures, likely due to matrix cracking around 60 °C. This behavior suggests a possible degradation of matrix integrity^30^ under thermal stress, despite initial stiffness gains.

The loss factor (tan δ) profiles (Figure 4b) exhibited two distinct peaks: the first around 30 °C, corresponding to the glass transition temperature (*T*_g_) of the PVB matrix,^30^ and the second, broader peak near 100 °C, associated with the glass transition of Kevlar.^45^ PPTA/PVB/GO and PPTA/PVB/MoS_2_ exhibited matrix *T*_g_ slightly higher than that of PPTA/PVB. This suggests that the polymeric chains in the PVB/GO and PVB/MoS_2_ matrices have lower mobility than those in the PPTA/PVB matrix,^20,43^ confirming the reinforcement effect.

Cole–Cole plots (Figure 4c) further supported these findings. In DMA, these plots (*E*″ vs *E*′) are sensitive to the number and nature of relaxation processes. Ideal semicircular arcs are typically associated with a single dominant relaxation and a more homogeneous viscoelastic response, whereas flattened or distorted arcs indicate overlapping relaxations and heterogeneity, often arising from interfacial polarization and restricted chain mobility near fiber–matrix contacts.^46–48^ The neat PPTA/PVB composite displayed a relatively narrow semicircular arc, suggesting a single dominant relaxation process with limited interfacial contribution. In contrast, the broader and less perfectly semicircular arcs observed for PPTA/PVB/GO and PPTA/PVB/MoS_2_ indicate the occurrence of additional interfacial relaxation mechanisms, typically associated with enhanced stress transfer and energy dissipation at the fiber/matrix interface. This improvement in energy dissipation could be particularly relevant in dynamic loading conditions such as ballistic impacts. In contrast, the c-PPTA/PVB/GO composite exhibited no notable change in the Cole–Cole plot, while c-PPTA/PVB/MoS_2_ showed an inconclusive behavior. These results highlight that matrix nanostructuration was a more efficient strategy than fiber surface deposition in enhancing interfacial synergy and energy dissipation mechanisms.

### Tensile Properties

The mechanical properties of the modified Kevlar composites were evaluated through tensile testing of single-ply samples.^49,50^ Figure 5 displays the representative stress–strain curves of the composites. The results reveal the typical stress–strain response of Kevlar composites as described by Zhu, Mobasher, and Rajan,^51^ featuring four distinct phases: the crimp region, the elastic region, the nonlinear failure region, and the postpeak region. Initially, in the crimp region, stress increases slowly as the yarns straighten by removing the inherent crimp from the weaving pattern. As the yarns become fully straightened and bear the load, the stress–strain curve steepens, transitioning into the elastic region, characterized by a linear load–displacement relationship. The slope of this linear region defines the Young’s modulus of the composites. The subsequent nonlinear failure region, which occurs before the ultimate tensile strength, is attributed to the random failure of individual filaments within the yarns. The stress abruptly decreases after reaching the ultimate tensile strength, indicating progressive yarn failure in the postpeak region. Notably, the hierarchical composites demonstrated superior performances compared to the PPTA/PVB composite.

**Figure 5:**
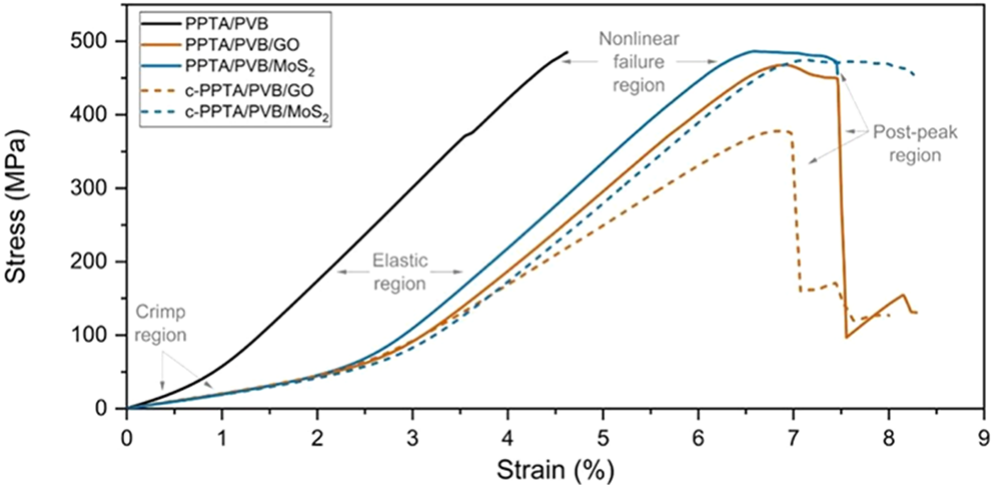
Representative stress–strain curves of the PPTA hierarchical composites.

The modulus at the crimp region is not usually measured, but the analysis became relevant as the hierarchical composites presented a much lower slope in this region. Figure 6a shows the modulus within the crimp region decreased from 3.8 ± 0.1 GPa in the PPTA/PVB composite to as low as 1.9 ± 0.1 GPa in the PPTA/PVB/MoS_2_ composite. Concurrently, the strain of the crimp region increased from approximately 0.5% in PPTA/PVB to 2.0% in the hierarchical composites. These changes suggest that the modified matrices allowed the fibers to straighten more efficiently before experiencing stress, without implying any increase in the initial crimp or irregularity of the fibers. A similar trend is observed for the Young’s modulus of the composites, with all hierarchicals showing a decrease, as shown in Figure 6b. However, only c-PPTA/PVB/GO showed an elastic modulus statistically different from that of the PPTA/PVB. Similarly, the hierarchical composites’ tensile strength was not significantly altered, except for c-PPTA/PVB/GO, as shown in Figure 6c.

**Figure 6:**
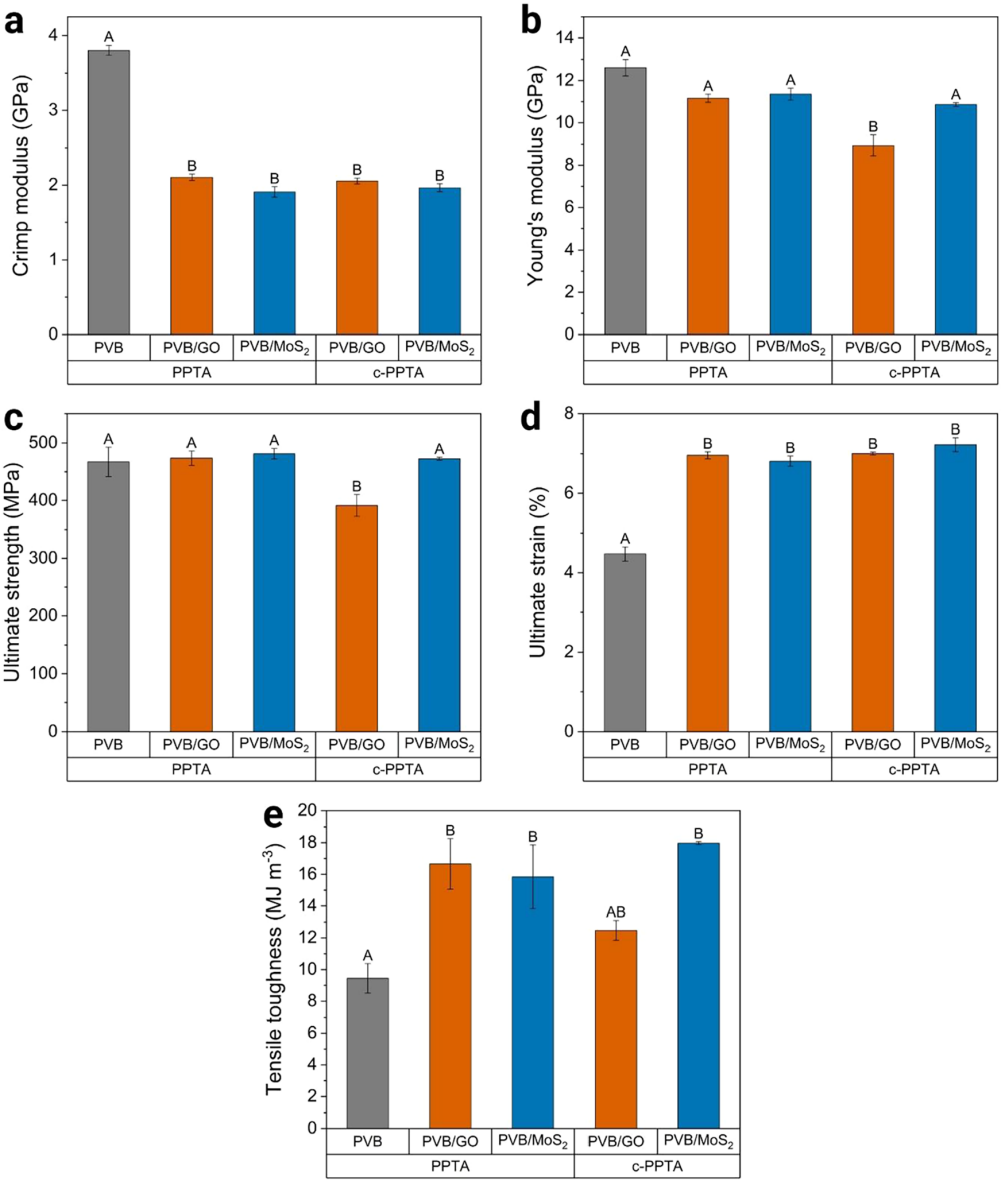
(a) Modulus at the crimp region, (b) Young’s modulus, (c) ultimate strength, (d) ultimate strain, and (e) tensile toughness of the hierarchical composites. Averages that do not share a letter are statistically different.

The observed stiffness and mechanical resistance loss of the hierarchical composites compared to the conventional PPTA/PVB was quite unexpected. According to the rule of mixtures, the modulus of an FRPC should be proportional to the moduli of the matrix and fiber.^52^ Therefore, as the PVB/GO and PVB/MoS_2_ matrices are stiffer than PVB,^30^ the hierarchical composites were expected to be stiffer than PPTA/PVB, as observed by many authors.^52–54^ However, the inapplicability of the rule of mixtures to the as-discussed composites may be related to the morphology of the composites resulting from the production processes, as proposed in the following section.

On the other hand, the hierarchical composites presented consistently higher ductility. As shown in Figure 6d, the ultimate strain of PPTA/PVB was 4.5 ± 0.2%, but increased to up to 7.2 ± 0.2% in the c-PPTA/PVB/MoS_2_ composite.

Consequently, the tensile toughness of the hierarchical composites, as calculated from the area under the stress–strain curve, was significantly enhanced. As shown in Figure 6e, PPTA/PVB/GO, PPTA/PVB/MoS_2_, c-PPTA/PVB/GO, and c-PPTA/PVB/MoS_2_ presented tensile toughness 76.4, 67.7, 32.1, and 90.3% higher than that of PPTA/PVB. Toughness enhancement in hierarchical composites may be associated with a matrix-fiber and fiber–fiber bridging effect promoted by the nanofillers^49^ as it avoids interyarn slippage and delamination, primary failure mechanisms in aramid fiber-reinforced composites. This effect can enhance interfacial adhesion and reduce fiber slippage, thereby increasing the load-carrying capacity of the FRPC. Additionally, it is suggested that the improved mechanical properties of Kevlar hierarchical composites could be linked to reduced residual stress and improved cohesive strength of the matrix.^50^

### Fractography

According to ASTM D 3039,^55^ failures in woven laminates can be classified using a standardized three-character code that describes failure type, area and location in the specimen, respectively. The first character identifies is the failure type: Angled (A), Edge delamination (D), Grip/tab (G), Lateral (L), Multimode (M), Longitudinal splitting (S), Explosive (X), Other (O); the second character is the failure area: Inside grip/tab (I), At grip/tab (A), <1 W from grip/tab (W), Gage (G), Multiple areas (M), Various (V), Unknown (U); and the third character is the failure location on the specimen: Bottom (B), Top (T), Left (L), Right (R), Middle (M), Various (V), Unknown (U). Together, this three-character code offers a concise and consistent description of the tensile specimen’s failure mode, helping to distinguish material responses and phenomena.

Figure 7 exhibits digital images of the area where the tensile failure occurred in the tested tapes. Interestingly, the composites exhibited composition-dependent failure modes, which correlated with their toughness, as materials with comparable toughness displayed analogous fracture patterns. The failure mode presented by the PPTA/PVB specimens (Figure 7a) was a lateral failure occurring in the middle of the specimen gage, classified as LGM. In the PPTA/PVB specimens, all the warp yarns broke in the same region, while the weft yarns remained almost intact. The toughest nanocomposites, namely, PPTA/PVB/GO (Figure 7b), PPTA/PVB/MoS_2_ (Figure 7c), and c-PPTA/PVB/MoS_2_ (Figure 7e), presented similar toughness also presented the same failure mode. The failure still occurred in the middle of the gage, but multiple types of failures could be identified, as not all the warp yarns broke, and some weft yarns were deformed, a typical MGM failure. c-PPTA/PVB/GO (Figure 7d), the composite with intermediary toughness, presented a third failure mode. In this case, a few warp yarns broke, but a considerable number of strained weft yarns can be observed, causing an angled failure at the top of the gage, or AGT failure. The weft yarn straining may be the reason for the lowest toughness achieved by this composite compared to the other hierarchical systems.

**Figure 7:**
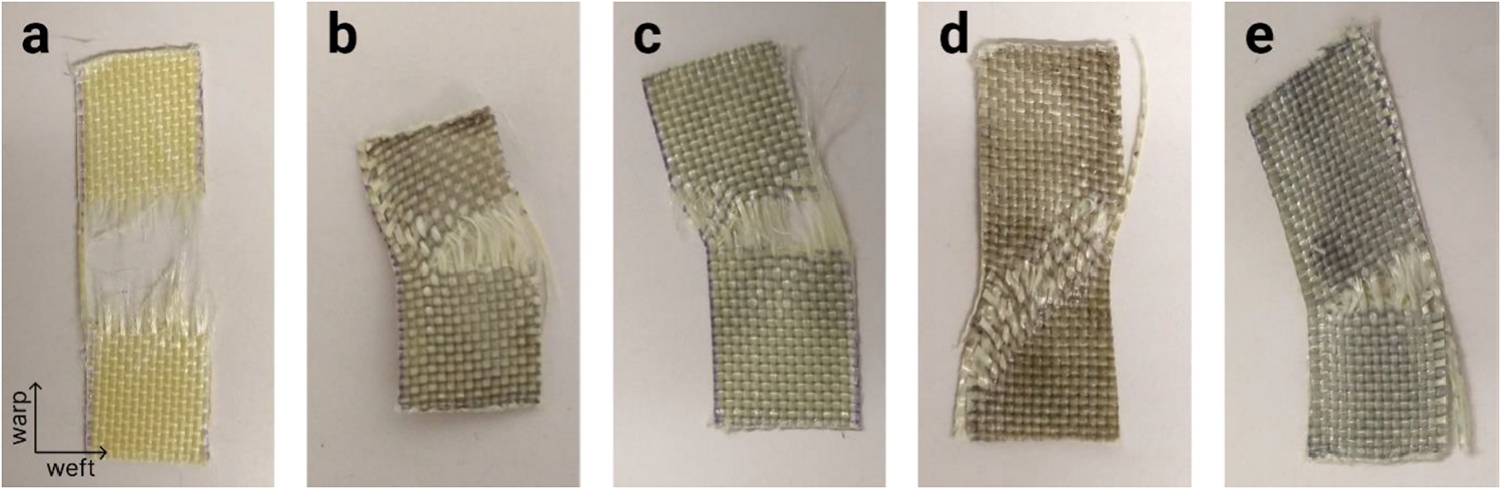
Digital photographs of the failure area in (a) PPTA/PVB, (b) PPTA/PVB/GO, (c) PPTA/PVB/MoS_2_, (d) c-PPTA/PVB/GO, and (e) c-PPTA/PVB/MoS_2_ hierarchical composite tapes.

Significant differences among the composites could be also noticed by analyzing SEM images of their fracture regions. Figure 8a,b shows that the neat PVB matrix presented a considerable plastic deformation, but its cracks, highlighted by yellow arrows, were mostly abrupt failures. In addition, a substantial number of broken fibers, highlighted by red arrows, are evident. In turn, Figure 8c reveals that the surface of most fibers exposed by the matrix failure had the aspect of raw fibers without matrix attachment. These features were the opposite of those observed in the hierarchical composites. Figure 8d,e shows that the matrix cracks in the PPTA/PVB/GO composite were much more plastic, and fewer fibers were broken. Besides, the matrix seemed to be much more attached to the fibers. Figure 8f shows that portions of the matrix clearly remain attached to the fibers’ surface, indicating a higher interfacial strength between the GO-modified matrix and the fibers. Similar conclusions can be taken from Figure 8g–i, related to the PPTA/PVB/MoS_2_ hierarchical composite. The matrix presented both plastic and fragile cracks, highlighted by yellow arrows in Figure 8g,8h, respectively. However, the fragile cracks could be related to matrix regions exhibiting poor MoS_2_ dispersion, as agglomerates, highlighted with green arrows in Figure 8h, were clearly visible near these cracks. Nonetheless, the enhanced fiber/matrix interaction can be noticed as portions of the matrix remained attached to the fibers, as is shown in Figure 8i.

**Figure 8:**
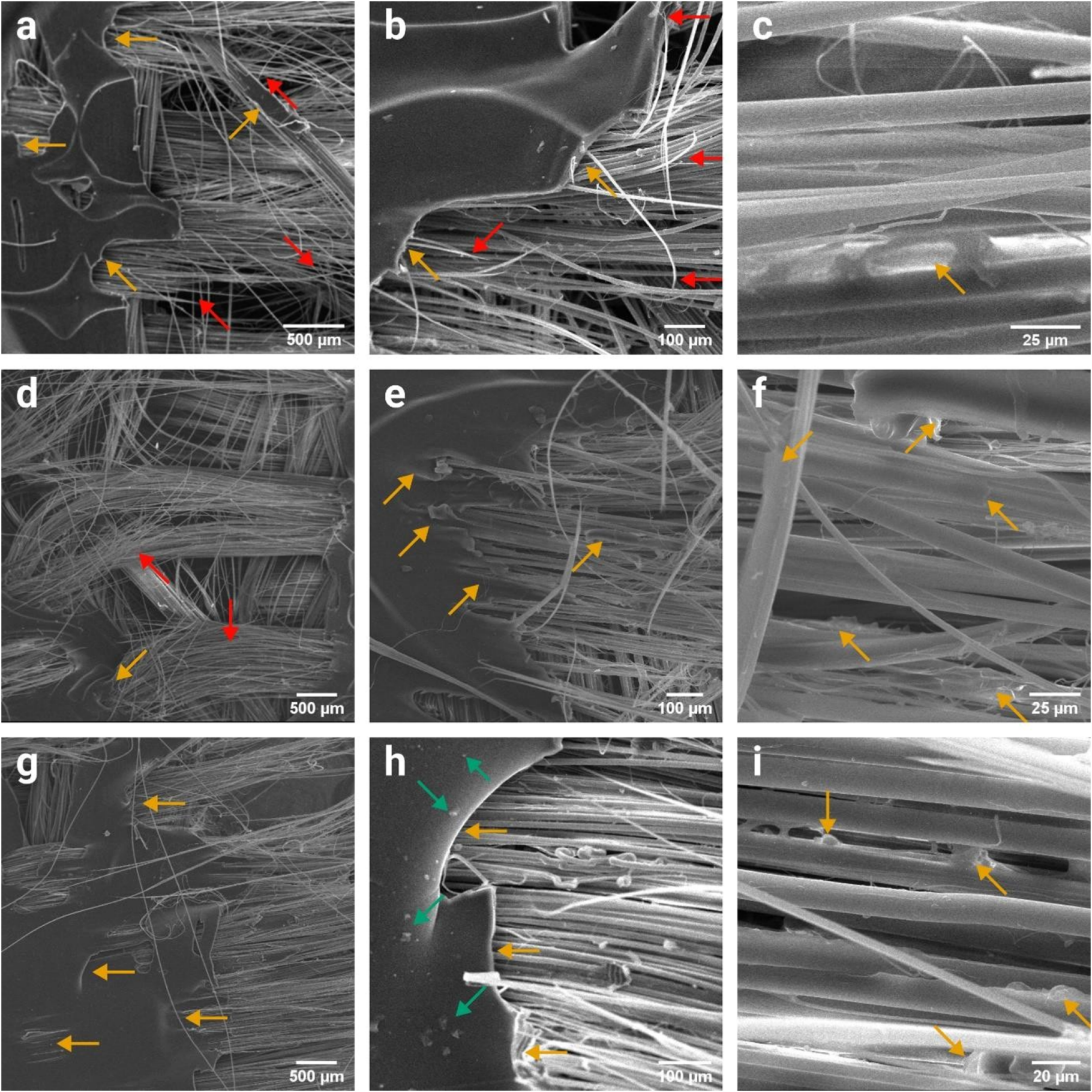
SEM images of the tensile failure region in the (a–c) PPTA/PVB, (d–f) PPTA/PVB/GO, and (g–i) PPTA/PVB/MoS_2_ composites. Yellow, red, and green arrows indicate points of interest regarding the matrix, fiber, and nanofiller, respectively.

The distinct failure behavior of the c-PPTA/PVB/GO composite was also evident in its fracture SEM images. As shown in Figure 9a, it exhibited fewer broken fibers compared to the previously analyzed composites. Instead of fiber breakage, tensile failure caused misalignment of the warp and weft yarns, leading to matrix cracking in multiple directions. Additionally, Figure 9b reveals fibrillation along the fiber diameter, a phenomenon known as peeling, promoted by fiber stretching, related to the existence of a gradient of properties inherent to the manufacturing process.^56^ This effect could be a direct consequence of fiber surface modification. Figure 9c shows a GO particle that apparently served as an anchoring point for fibrillation deposited on the fiber surface.

**Figure 9:**
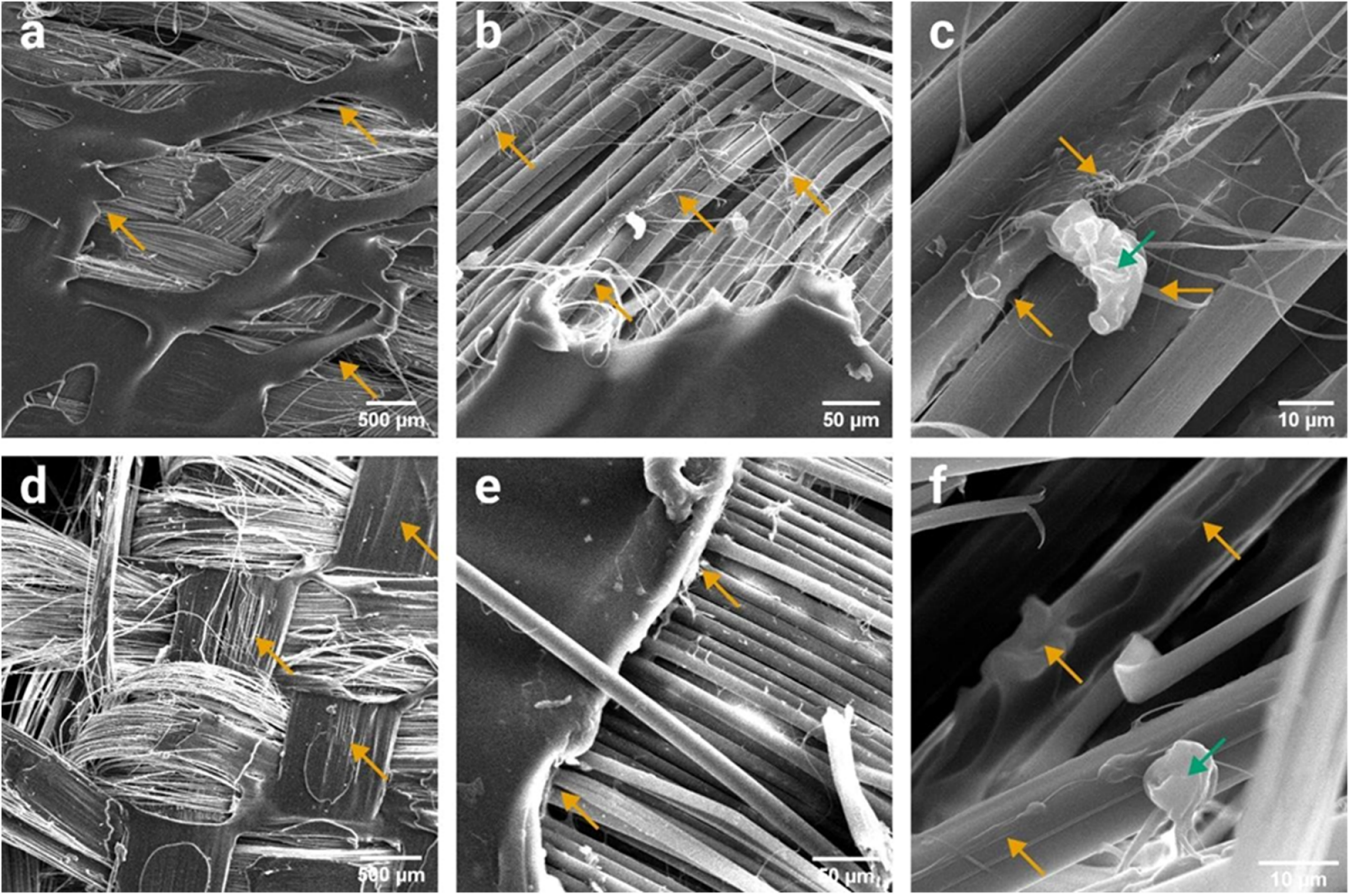
SEM images of the tensile failure region of the (a–c) c-PPTA/PVB/GO, and (d–f) c-PPTA/PVB/MoS_2_ composites. Yellow and green arrows indicate points of interest regarding the matrix and nanofiller, respectively.

On the other hand, the c-PPTA/PVB/MoS_2_ composite presented a failure mechanism that was not observed in the other systems. The yellow arrows in Figure 9d highlight the areas where the weft yarns caused matrix splitting, i.e., local cracks or scissions in the polymer matrix along the fiber orientation induced by yarn architecture and stress concentration during failure.^57^ In addition, despite the fragile failure of the matrix, which is shown in Figure 9e, a considerable portion remained attached to the fibers, as can be seen in Figure 9f, evidencing good adhesive cohesion between the fiber and the matrix.

Therefore, the nanofillers’ deposition on the fiber’s surface apparently led to higher fiber/matrix adhesion. However, the higher adhesion increased the weft yarns’ participation in the resistance mechanisms, creating a competing process that prevented further enhancement of the composites’ mechanical properties.

SEM images also revealed the probable reason behind the inapplicability of the rule of mixtures to the aramid composites. Noticeably, the composites’ matrix was just a film that covered the textile surface. Thus, the inner yarns had no contact with the matrix. This structure is extremely different from that observed in carbon fiber-reinforced epoxy, for example, in which the matrix involves each fiber individually, resulting in efficient stress transfer and estimating the composite’s Young’s modulus according to the rule of mixtures.^52,58^ Even though this impregnation issue could be theoretically solved during compression molding, where temperature and pressure would force the polymer to flow and reach the inner side of the fabric, at the working temperature, the formation of H-bonding occurs in PVB, increasing the chain interlocking and the polymer viscosity, preventing its flow.^59^

As the differences presented by the nanofiller-covered composites, compared to the matrix-modified composites, were not that expressive, PPTA/PVB/GO and PPTA/PVB/MoS_2_ composites were selected to undergo further ballistics tests.

### Body Armor Ballistics Tests

Assessment of the body armor ballistic performance considered 4 features: number of perforated Kevlar plies, back face signature (BFS), specific energy absorption (SEA) and the ballistic limit. The number of perforated plies indicates how many layers of the ballistic vest are penetrated by a projectile during testing. It provides a direct measure of the vest’s resistance and helps assess the likelihood of complete penetration or failure. Back face signature (BFS) refers to the maximum deformation on the rear (body-facing) surface of body armor upon projectile impact, measured using a calibrated backing material. It quantifies the blunt trauma transmitted to the wearer even when the armor prevents penetration. BFS is critical for evaluating armor performance, as excessive deformation can cause serious internal injuries despite stopping the bullet. Lower BFS values are important in body armor as they indicate reduced blunt trauma and better protection for the wearer, translating to enhanced overall safety.^31^

The first analysis of the ballistics tests considered only the shots with an impact velocity within the range specified by the NIJ 0101.04 standard, i.e., 341 ± 9 m s^–1^.^31^ This was crucial for comparing the different composites under similar testing conditions. Figure 10a shows that, at similar impact velocities, there were no significant differences in the number of perforated plies among the distinct composites. However, the back face signature of PPTA/PVB/GO and PPTA/PVB/MoS_2_ were reduced by 3 and 7%, respectively, compared to PPTA/PVB.

**Figure 10:**
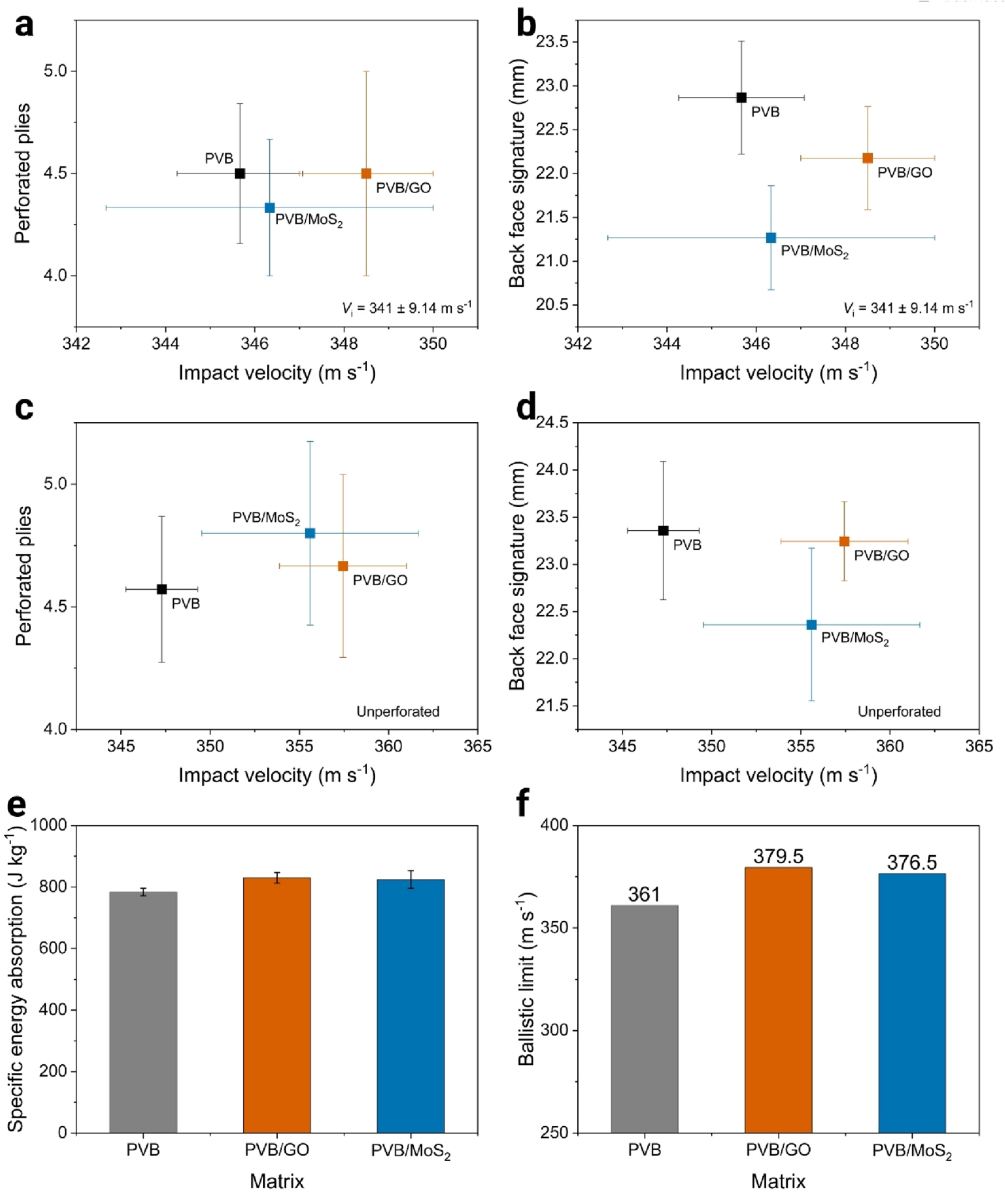
(a) Number of perforated Kevlar plies and (b) back face signature for each composite as a function of shot impact velocity within the NIJ 0101.04 standard range. (c) Number of perforated Kevlar plies and (d) back face signature as a function of impact velocity for all retained shots. (e) Specific energy absorption for each composite based on all shots retained by the ballistic plates. (f) Ballistic limit of Kevlar composites with different matrices.

When analyzing the shots retained by the ballistic plaques, i.e., those that did not fully perforate the armor, additional insights emerge. Hierarchical composites could retain projectiles with velocities approximately 10 m s^–1^ higher than those maintained by the PPTA/PVB composite. Nonetheless, the projectiles did not penetrate beyond 5 Kevlar plies, as illustrated in Figure 10c. Moreover, even with higher-velocity projectiles, the back face signature of the PPTA/PVB/GO composite was not greater than that of the PPTA/PVB composite and was lower in the PPTA/PVB/MoS_2_ composite, as shown in Figure 10d.

To enable a more accurate comparison of composite performance, the specific energy absorption for each shot was calculated using eq 1, where *m*_p_ and *m*_A_ are the mass of the projectile and the armor, respectively, and *u*_imp_ is the impact velocity.^4^

1 $$\mathrm{SEA}=\frac{\left(1/2\right)m_{p}u_{\mathrm{imp}}^{2}}{m_{A}}$$

Figure 10e shows that SEA of the hierarchical composites was higher than that of the neat composite. Specifically, PPTA/PVB had an SEA of 783 ± 11 J kg^–1^, while PPTA/PVB/GO and PPTA/PVB/MoS_2_ achieved SEAs of 829 ± 17 and 824 ± 29 J kg^–1^, respectively. SEA is a direct measurement of the material’s ability to absorb and dissipate the energy from high-velocity impacts. Higher SEA means that the 2D nanoparticle reinforced composites can absorb more energy before failing, thereby reducing the exit velocity of the projectile and mitigating damage to the underlying structure or body.^4^

The final analysis of the ballistic tests focused on determining the ballistic limit (*V*_50_) of each composite. According to the NIJ methodology, *V*_50_ is defined as the arithmetic mean between the highest velocity at which no complete penetration occurred and the lowest velocity at which complete penetration was observed.^60^ As shown in Figure 10f, the neat PPTA/PVB composite exhibited a *V*_50_ of 361 m/s, while the incorporation of GO and MoS_2_ increased this value to 379 and 376 m/s, respectively. These results correspond to performance improvements of approximately 5% compared to the reference material.

Noticeably, despite the distinct chemical and morphological features of GO and MoS_2_, as well as their resulting dispersion efficiency within the matrix^30^ and in the composites, as evidenced by SEM images, PPTA/PVB/GO and PPTA/PVB/MoS_2_ composites showed similar overall mechanical and ballistic performance. This outcome may result from a combination of factors, including the dominant contribution of the PPTA/PVB matrix to the overall response, the possibility that different reinforcement mechanisms provided by GO or MoS_2_ balance each other at the macroscopic scale, and the influence of heterogeneous dispersion, which can limit the maximum efficiency of both nanomaterials.

Nonetheless, the enhancements of 6% in SEA, and 5% in the ballistic limit indicate that the nanostructuration of the PVB matrix with GO and MoS_2_ could improve the performance of aramid body armors. These results are comparable to those from authors who also employed nanoadditives in the matrix of ballistic protection composites. Manero II et al.^61^ reported that the addition of 1.0 wt % of carbon nanotubes was capable of increasing the *V*_50_ of a Kevlar thermoset system by 7.3%. Pol, Liaghat, and Hajiarazi^62^ saw an increase of nearly 5% in the energy-absorbing capability of E-glass/epoxy laminated composites when 5 wt % nanoclay was incorporated into the composite. Rahman et al.^63^ achieved about 6% of enhancement of the ballistic limit by adding 0.3 wt % of multiwalled carbon nanotubes into E-glass/epoxy composites.

Thus, these results may be considered as promising, however, further work on the many parameters related to the construction of ballistic protection devices and applications could improve the hierarchical composites. For example, an impregnation method that enhances matrix diffusion through the fabric could further improve performance by increasing fiber wetting. In the case of PVB/MoS_2_, better nanofiller dispersion^30^ in the matrix could be achieved through functionalization, leading to higher performance. Further analyses to clarify the effects of the nanofillers on the fiber/matrix interface may enable improvements in the c-PPTA composites. Thus, the nanostructuration of the matrix can yield relevant results so that the weight of the armors can be reduced by eliminating one or more Kevlar plies in the future.

In addition, the mechanical and dynamic mechanical properties of PPTA/PVB/GO and PPTA/PVB/MoS_2_ composites were comparable to those reported by Obradović et al.^20^ for PPTA/PVB composites reinforced with other nanomaterials. Although these authors did not evaluate the ballistic protection level of their hierarchical composite, they reported up to 48% greater energy absorption after knife penetration compared to PPTA/PVB. This suggests that PPTA/PVB/GO and PPTA/PVB/MoS_2_ composites could still be applied in other types of armors, providing significantly superior performances compared to the currently available solutions.

Finally, one may wonder why the remarkable enhancements, such as increases of up to 90% in ductility and tensile toughness compared to the original PPTA/PVB composite, resulted in an increment of only 5% in the ballistic limit. This discrepancy can be largely attributed to the extremely different strain rates applied in quasi-static tensile tests and ballistic impacts, as materials may exhibit entirely distinct mechanical responses under different strain rates. The dynamic response of PPTA/PVB systems is expected to be rate-sensitive due to both the strain-rate dependence of aramid fibers and the viscoelastic nature of the PVB matrix.^64,65^ Previous studies show that aramid fibers typically exhibit increased tensile strength at higher strain rates while their elastic modulus is comparatively less affected,^66^ and that PVB presents pronounced relaxation and damping behavior that depends on loading rate and temperature.^67,68^ Consequently, the macroscopic ballistic response results from the combined rate-dependent contributions of fiber, matrix and interface, and cannot be fully inferred from quasi-static or low-rate DMA alone. To bridge this gap, instrumented drop-weight impact tests^65^ and split-Hopkinson experiments^66,69^ on single plies or thin laminates have been used in the literature to probe intermediate and high strain-rate regimes and to identify transitions in dominant failure mechanisms. Although such high-rate tests were not performed in the present work, they are recommended as follow-up studies to quantitatively link the DMA results to the mechanical response at ballistic strain rates.

## Technological Outlook

The proposed approach also presents favorable prospects for industrial scalability. Both nanomaterials employed in this study, i.e., GO and MoS_2_ are commercially available, with graphene oxide being the most accessible and already offered in application-tailored formulations in commercial scale. The scalability of graphene derivatives has significantly advanced over the past decade, leading to cost reductions and broader market availability.^70–72^ The additional cost of incorporating nanomaterials into ballistic plates is justified by the superior performance of the hierarchical composites, which ultimately translates into enhanced protection and potential life-saving benefits. Moreover, future developments in nanotechnology may enable further performance improvements, possibly reducing the number of Kevlar layers required, thereby lowering both cost and weight of armor systems. From a processing standpoint, the dip-coating and impregnation procedures adopted here are consistent with methods already used at industrial scale. In practice, the industrial fabrication of PVB-based prepregs involves dissolving PVB pellets or scraps in ethanol, a step in which nanomaterials can be readily incorporated without altering subsequent processing stages of ballistic vest production. Finally, health, safety, and environmental aspects related to nanomaterials must also be considered. However, extensive research efforts^73,74^ are constantly establishing reliable parameters and standardized guidelines, aiming to ensure their safe handling and sustainable integration into industrial processes.

## Conclusions

Hierarchical aramid composites were successfully developed using either graphene oxide (GO) or molybdenum disulfide (MoS_2_) as nanomodifiers in the poly­(vinyl butyral) matrix or as surface coatings on Kevlar fibers.

Dynamic mechanical and tensile analyses demonstrated that matrix nanostructuration, rather than fiber coating, was the most effective approach, increasing storage modulus, enhancing interfacial adhesion, and improving energy dissipation. All nanomodified systems exhibited markedly higher ductility and tensile toughness (up to 90% greater than the neat composite), confirming superior load transfer and failure resistance mechanisms.

Ballistic testing of level II-A armor plates revealed tangible performance gains: specific energy absorption and ballistic limit increased by 6% and 5%, respectively, accompanied by reduced back-face signature. These results indicate that incorporating 2D nanomaterials into PVB matrices can effectively enhance the protective efficiency of aramid composites without increasing weight.

Overall, the findings validate matrix nanostructuration with GO or MoS_2_ as a practical and scalable strategy for next-generation lightweight body armor.

## ⊥

Department of Mechanical Engineering, École de Technologie Supérieure, 1100 Notre-Dame St W, Montreal, Quebec H3C1K3, Canada

## #

DuPont, Av. Sylvio Honório Álvares Penteado, 370, Tamboré, Barueri, SP 06460–025, Brazil

## ∇

Department of Chemical Engineering, Polytechnique Montréal, 2500 Chemin de la Polytechnique, Montréal, Quebec H3T 1J4, Canada
